# Supplementary material for: Genomic analyses reveal the stepwise domestication and genetic mechanism of curd biogenesis in cauliflower
Source: Nat Genet. 2024 May 7;56(6):1235–44. doi: 10.1038/s41588-024-01744-4 (PMC11176064; doi:10.1038/s41588-024-01744-4)
Supplement: Supplementary file 1 — Supplementary Figs. 1–13. [file 41588_2024_1744_MOESM1_ESM.pdf]

nature genetics

Article

https://doi.org/10.1038/s41588-024-01744-4

Genomic analyses reveal the stepwise domestication and genetic mechanism of curd biogenesis in cauliflower

In the format provided by the authors and unedited

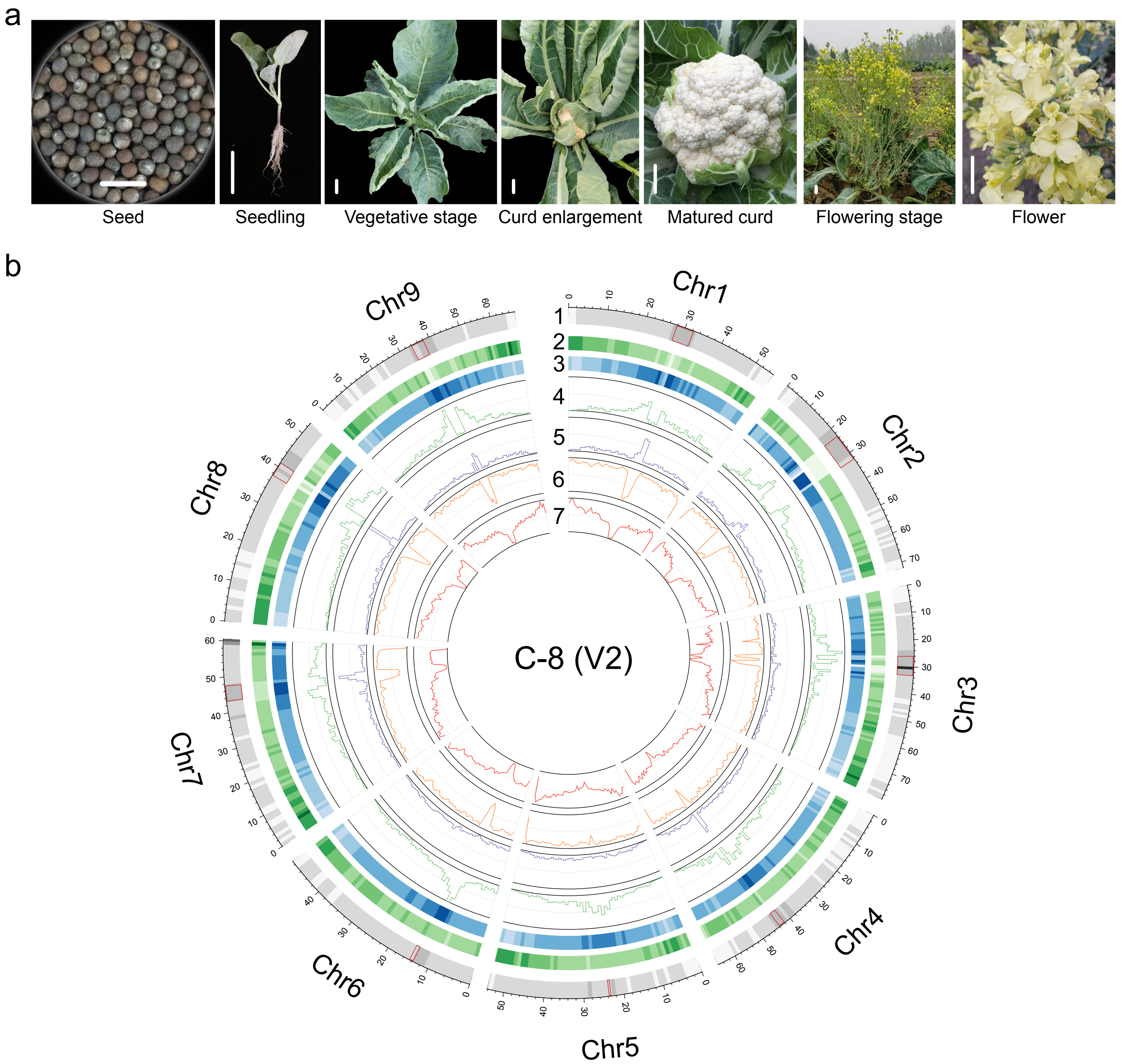

Supplementary Figure 1. Overview of the cauliflower genome assembly C-8 (V2). a, Photos of cauliflower C-8. White scale bars: 5 mm in seed, 5 cm in others. **b**, Circos plot of chromosomal distributions of genomic attributes. The circles, from outside to inside, correspond to GC content (1, gray heatmap), gene density (2, green heatmap), repeat sequences (3, blue heatmap), and densities of Gypsy-type (4, green lines) and Copia-type (5, purple lines) LTRs, as well as SNPs (6, orange lines) and InDels (7, red lines). Red rectangles indicate putative centromeric regions. Window size is 1 Mb.

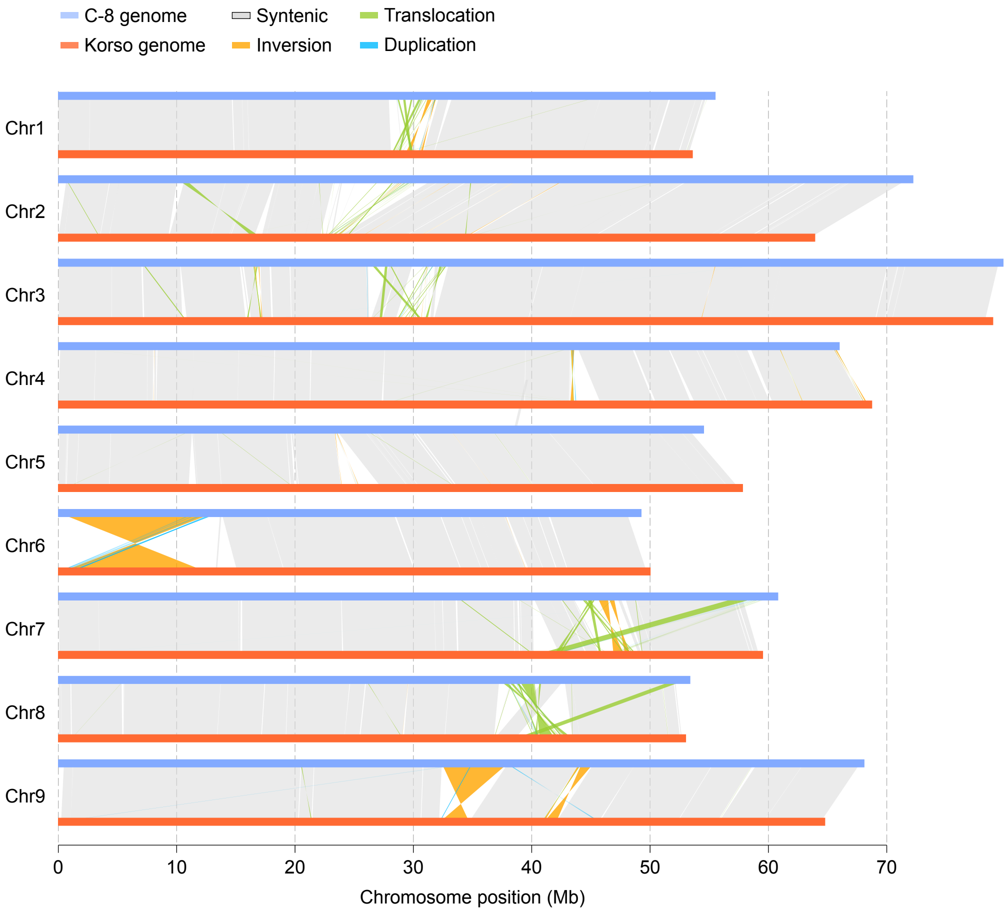

Supplementary Figure 2. Collinearity and structural variants (inversions, translocations and duplications) between C-8 (V2) and Korso reference genomes.

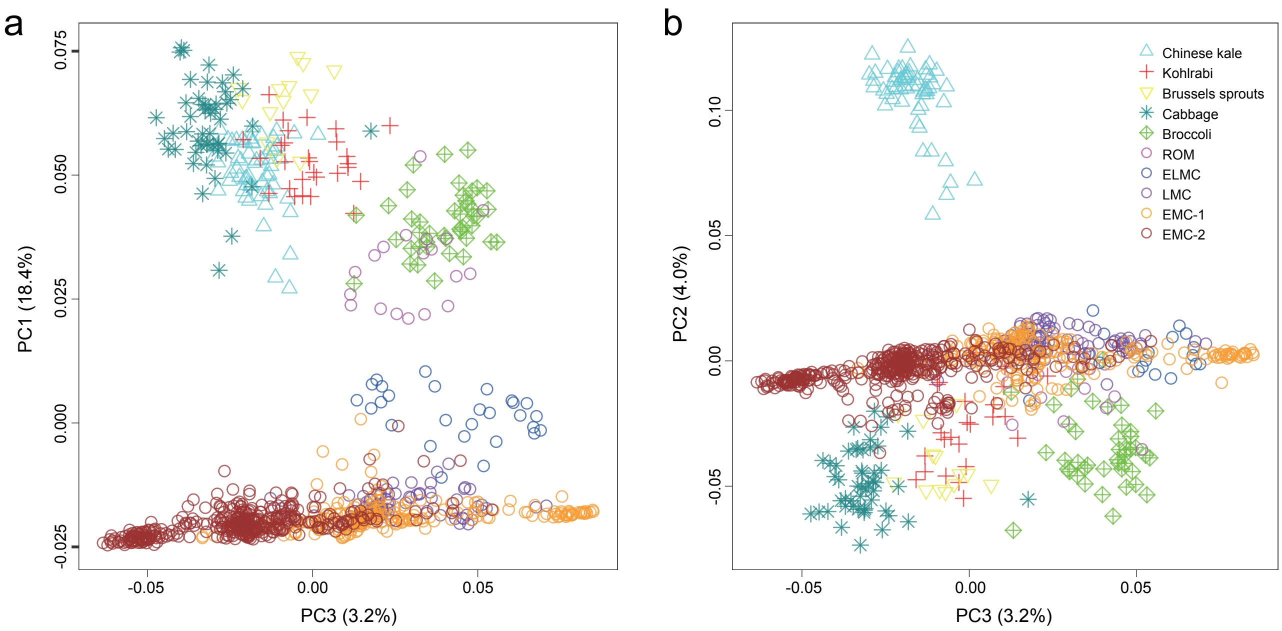

Supplementary Figure 3. PCA analysis based on 1,564 4d-SNPs for PC1 versus PC3 (a) and PC2 versus PC3 (b).

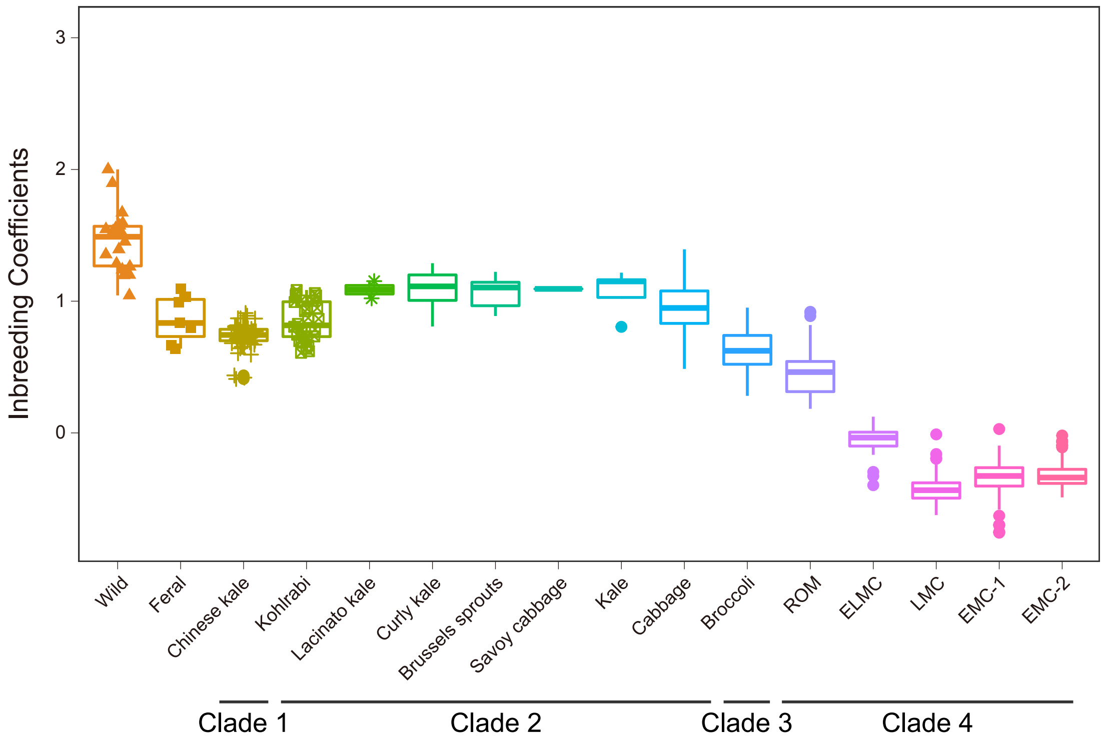

Supplementary Figure 4. Statistics of inbreeding coefficients in wild and cultivated groups. For each group, the sample size was set as follows: Wild (n=18), Feral (n=7), Chinese kale (n=62), Kohlrabi (n=28), Lacinato kale (n=2), Curly kale (n=5), Brussels sprouts (n=13), Savoy cabbage (n=2), Kale (n=5), Cabbage (n=49), Broccoli (n=43), ROM (n=22), ELMC (n=28), LMC (n=95), EMC-1 (n=224), and EMC-2 (n=360) (centerline, median; box limits, first and third quartiles; whiskers, 1.5 × IQR).

.

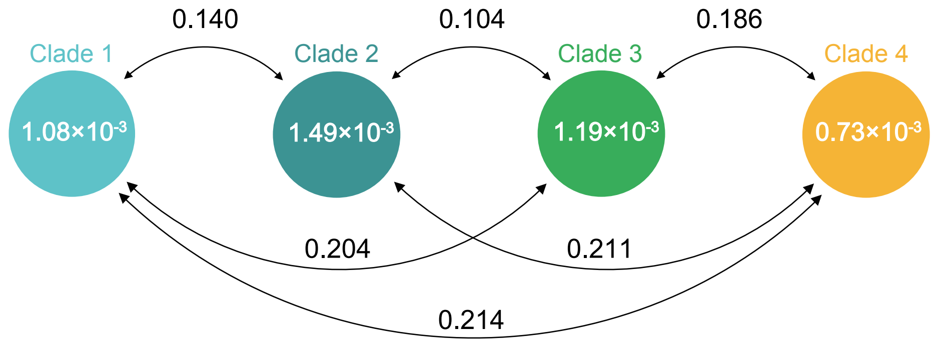

Supplementary Figure 5. Summary of nucleotide diversity (π) and population divergence (F_{ST}) among four clades.

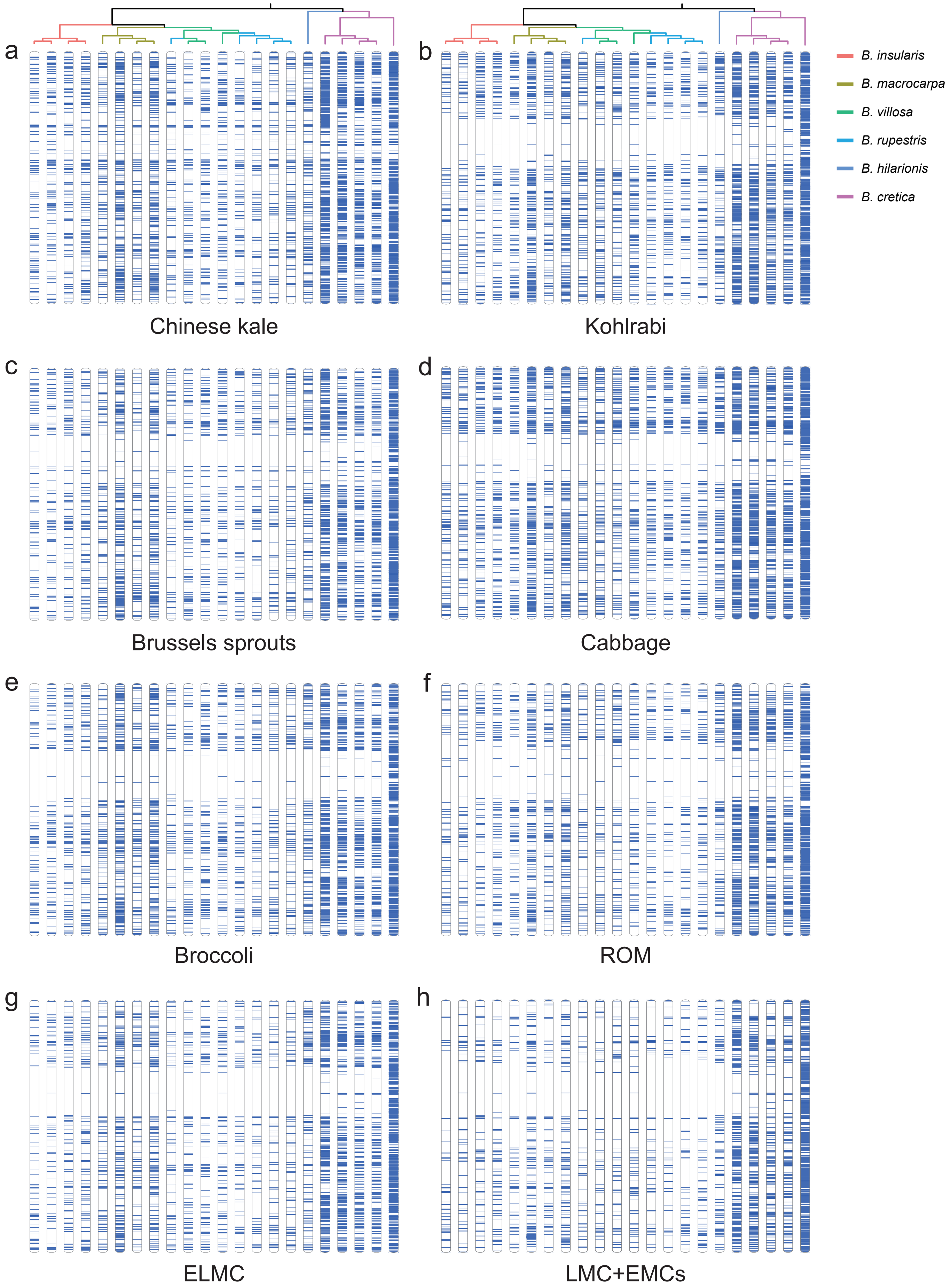

Supplementary Figure 6. Diagram of inferred syntenic regions between each group of *B. oleracea* and 22 wild accessions in chromosome 3.

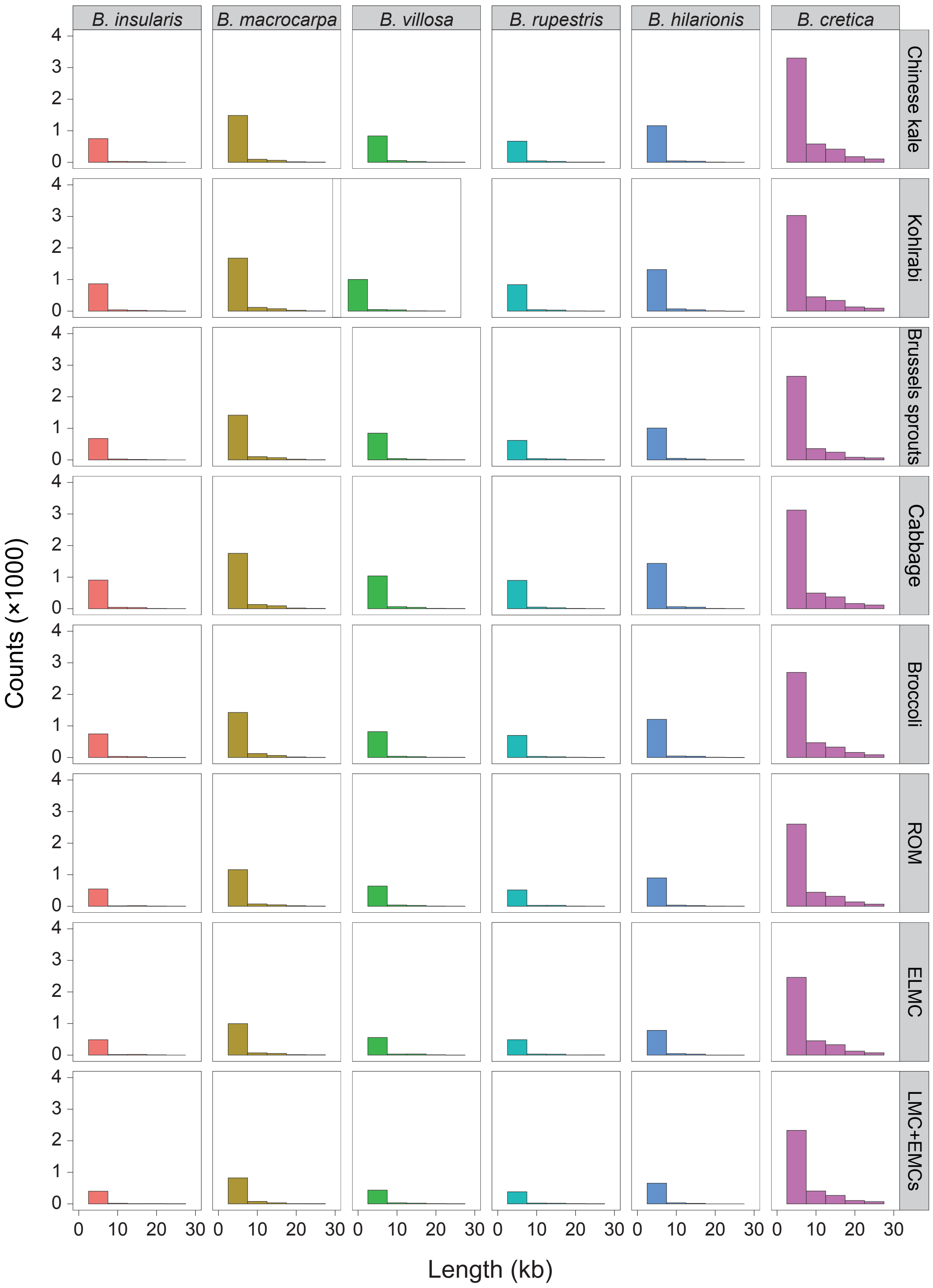

Supplementary Figure 7. Histograms of identical fragments between each group of *B. oleracea* and six wild species. A single representative accession was selected for each wild species as follow: SRR6453800, *B. insularis*; C0_0166, *B. macrocarpa*; SRR6453822, *B. villosa*; SRR6453618, *B. rupestris*; SRR6453871, *B. hilarionis*; SRR9331105, *B. cretica*.

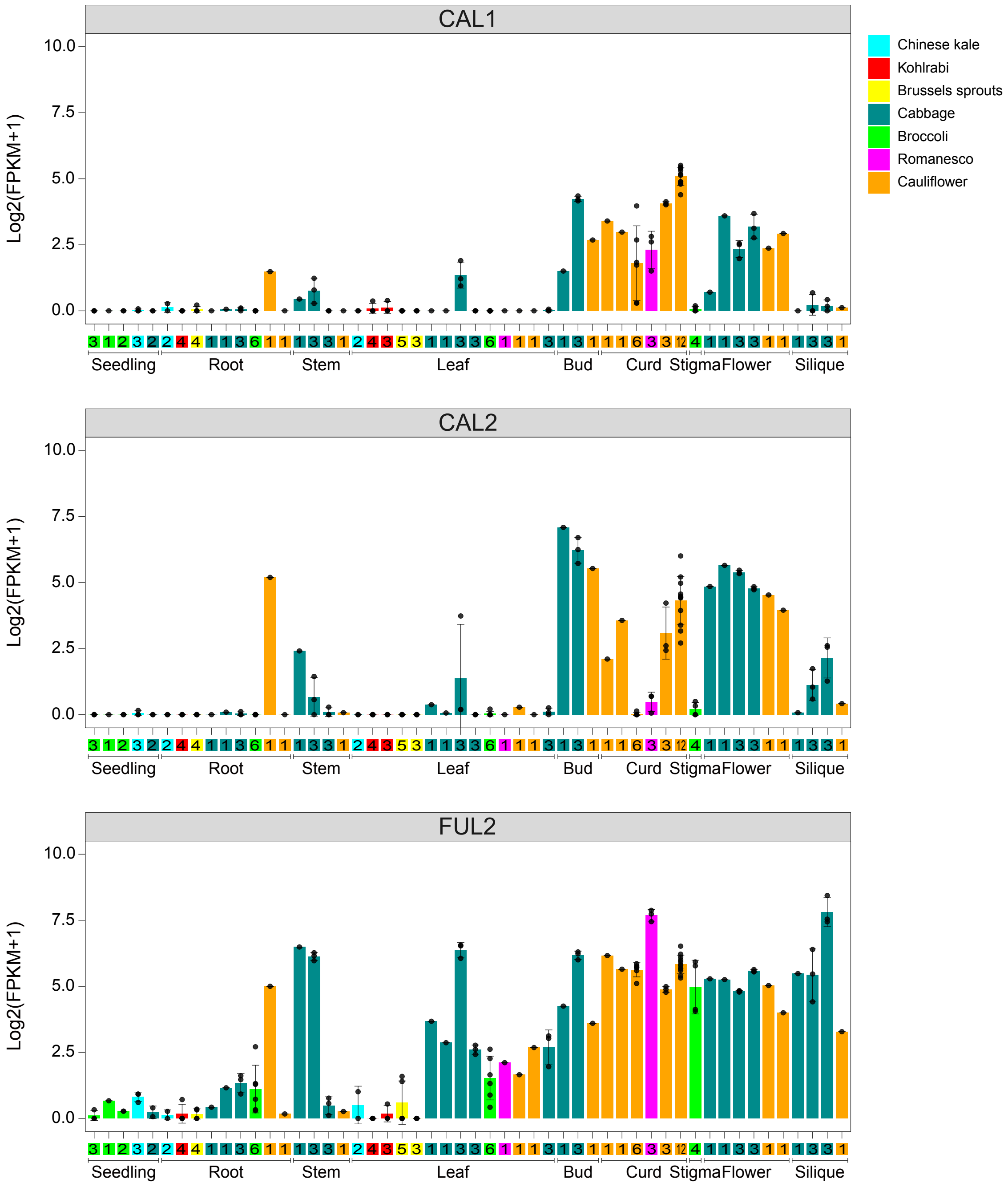

2.5

Supplementary Figure 8. Tissue-specific transcriptome analyses of *CAL1*, *CAL2* and *FUL2* genes.
For each category, the gene expression level was presented as mean ± standard deviation of log2(FPKM+1). The sample size was indicated by the number located in the underlying squares.

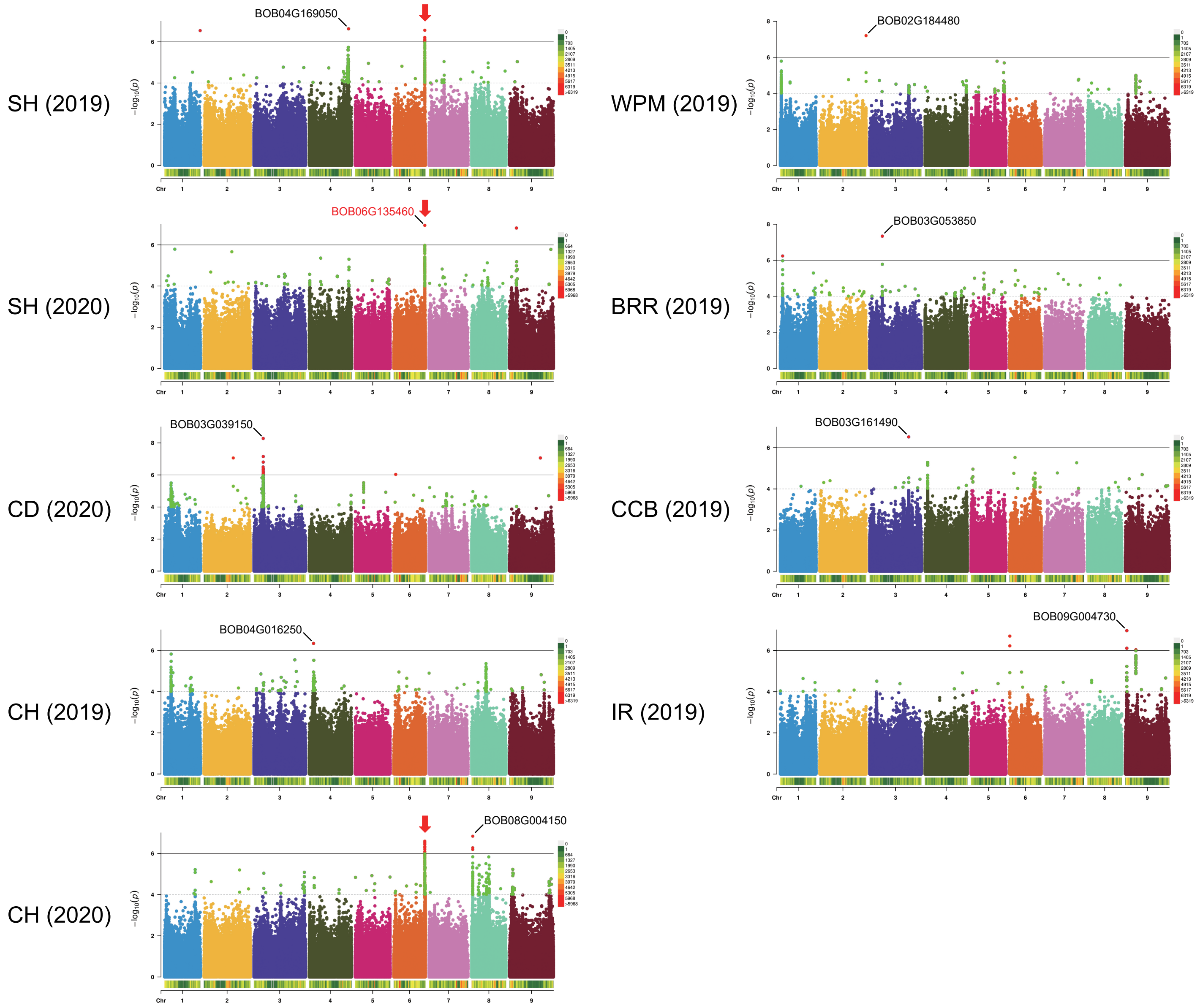

Supplementary Figure 9. Manhattan plots and candidate genes of seven important agronomic traits in cauliflower. The red arrows indicate identical signals. SH, stem height; CD, curd diameter; CH, curd height; WPM, whole-plant mass; BRR, black rot resistance; CCB, color of curd branch; and IR, insect resistance.

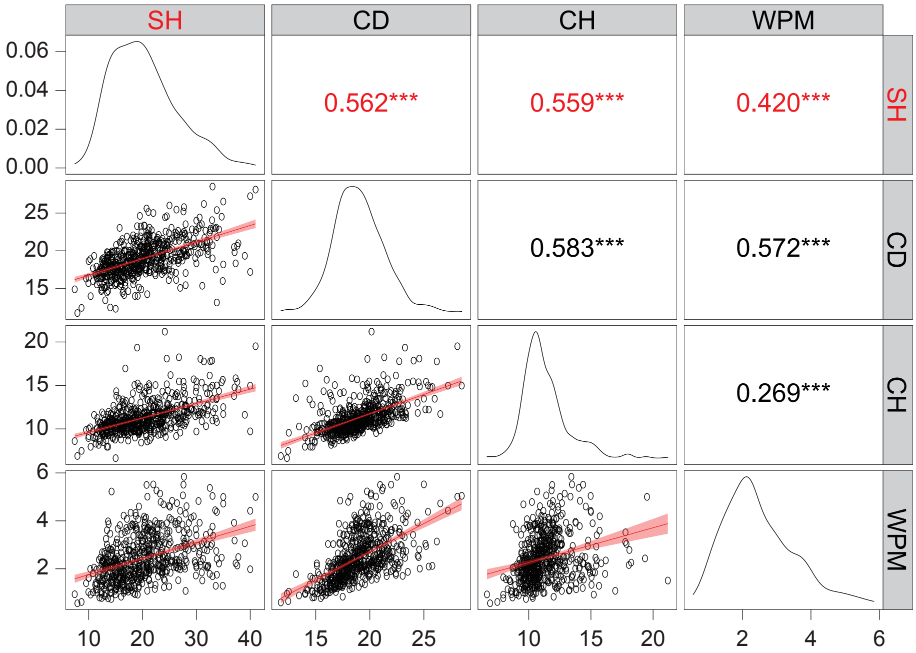

Supplementary Figure 10. Phenotype correlation analyses of SH, CD, CH, and WPM. Asterisks indicate significance of the correlations as *** $p < 0.001$ (Spearman). Red lines and regions in the lower diagonal are based on linear regressions and the 95% confidence intervals.

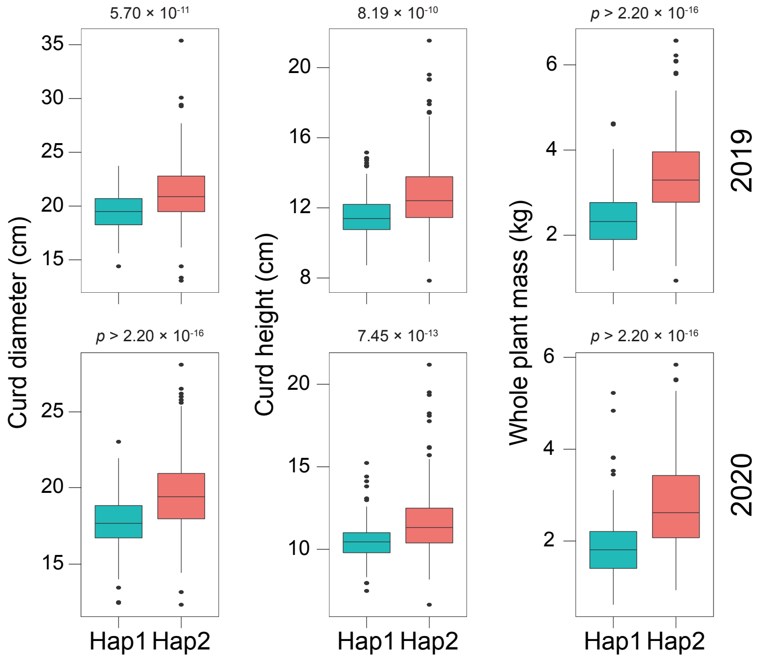

Supplementary Figure 11. Proportion of different haplotypes existing in CD, CH, and WPM in 2019 and 2020. Statistical significance was determined by two-sided Student's t-tests. The sample size is n_{Hap1}=125, n_{Hap2}=244 in 2019, and n_{Hap1}=172, n_{Hap2}=391 in 2020 (centerline, median; box limits, first and third quartiles; whiskers, 1.5 × IQR).

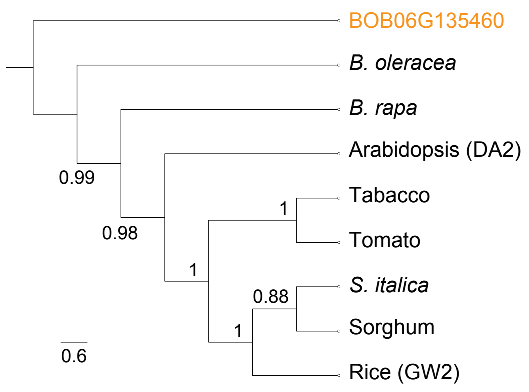

Supplementary Figure 12. Phylogenetic tree of *BOB06G135460* and its orthologs across nine monocot and dicot species.

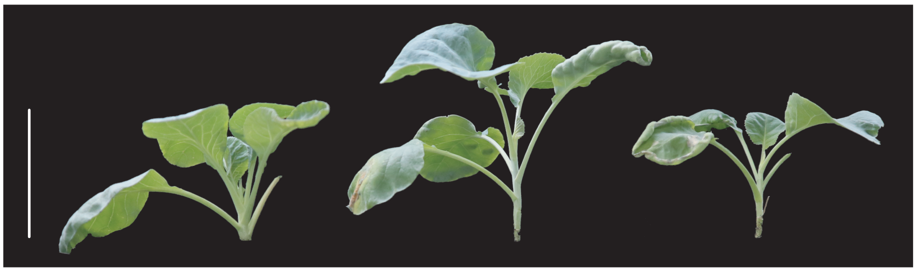

CK OE-1 OE-2

Supplementary Figure 13. Plant morphologies of overexpressing *BOB06G135460* in cauliflower. White bar indicates 5 cm.
